# Effectiveness of Ceftriaxone Treatment in Preventing Relapse-like Drinking Behavior Following Long-term Ethanol Dependence in P Rats

**DOI:** 10.4172/2155-6105.1000183

**Published:** 2014-04-30

**Authors:** PSS Rao, Y Sari

**Affiliations:** Department of Pharmacology, College of Pharmacy and Pharmaceutical Sciences, University of Toledo, Toledo, OH, USA

**Keywords:** Glutamate, GLT1, Relapse, P rats, Ceftriaxone

## Abstract

**Objective:**

To evaluate the effectiveness of ceftriaxone treatment in attenuating relapse-like ethanol drinking behavior in male P rats following 14-weeks of continuous ethanol consumption.

**Methods:**

After 14-weeks of continuous access to free choice of 15% and 30% ethanol, male P rats were deprived of ethanol for two weeks. On the last five days of abstinence period, P rats were treated, once a day, with either saline or ceftriaxone (50 or 200 mg/kg; i.p.). This was followed by re-exposure to ethanol for the next 10 days to simulate the relapse-like ethanol drinking behavior.

**Results:**

Ceftriaxone treatment (during abstinence) reduced ethanol intake upon re-exposure to ethanol, compared to the saline treated P rats. This statistically significant reduction in ethanol consumption in P rats following treatment with ceftriaxone (200 mg/kg/day) was observed from Day 2 to Day 9. Similarly, water consumption in P rats treated with ceftriaxone was significantly higher than the saline treated group between Day 2 and Day 7. Importantly, ceftriaxone treatment at both doses did not cause any significant changes in body weight compared to saline treated group.

**Conclusions:**

We report here that ceftriaxone at higher dose has been found to be effective in the attenuation of relapse-like ethanol-drinking behavior in chronic ethanol intake model. This is in accordance with previous data from our lab in cocaine animal model demonstrating that only higher dose of ceftriaxone has been effective in attenuating cocaine relapse.

## Introduction

Relapse rates for addiction were found to be similar to those of chronic illness such as hypertension, asthma, diabetes [1]. Various factors, external and internal, have been associated with relapse to drug-seeking and drug-consumption behaviors [2–5]. Much attention has been focused for studying relapse to ethanol consumption following a period of abstinence [6–10]. Several factors have been attributed to this resumption of ethanol consumption [11–13]. Subsequently, studies have revealed multiple targets/biomarkers for the prediction and treatment of alcohol relapse [14,15].

The significance of glutamatergic system in development of alcohol dependence has been extensively studied [16]. Ethanol has been suggested as an inhibitor of the N-Methyl-D-Aspartate (NMDA) receptors and chronic exposure to ethanol results in increased expression of these receptors [17–19]. Interestingly, withdrawal from alcohol results in a hyperexcitability state which has been implicated in withdrawal related seizures and neuronal cell death [20–22]. Apart from influencing the expression of the NMDA receptors, we have previously reported the role of the major glutamate transporter, GLT1, in the nucleus accumbens (NAc) [23].

Ceftriaxone was found effective in preventing relapse to cocaine seeking in rats [24,25]. Furthermore, we have recently demonstrated the effectiveness of ceftriaxone treatment in reducing ethanol intake by ethanol-dependent alcohol-preferring (P) rats via modulation of GLT1 expression in both NAc and prefrontal cortex (PFC) [23,26]. Moreover, since relapse to ethanol drinking has been linked to the increased glutamate neurotransmission in the mesocorticolimbic reward pathway, we also have evaluated the efficacy of ceftriaxone treatment in relapse-like drinking paradigm. Five weeks of free-choice ethanol exposure was followed by a two-week abstinence period [27].

While previous work concerning efficiency of ceftriaxone treatment in relapse-like ethanol- drinking behavior proved effective following five-weeks of exposure to ethanol, our aim for the present study was to evaluate the therapeutic value of ceftriaxone treatment in relapse-like ethanol-drinking by P rats following 14-weeks of ethanol dependence. After 14-weeks of continuous access to free choice of 15% and 30% ethanol solutions, P rats were deprived from ethanol for 2 weeks. On the last 5 days of this abstinence period, P rats were treated, daily once, with either saline or ceftriaxone (50 or 200 mg/kg; i.p.). This was followed by re-exposure to ethanol for the next 10 days to simulate the relapse-like ethanol-drinking behavior.

## Materials and Methods

### Animals

Male P rats were obtained from the Indiana University School of Medicine (Indianapolis, IN) breeding colonies. Animals, single housed in wood-chip bedded plastic cages, were housed in a temperature (21°C) and humidity (50%) controlled vivarium maintained on a 12/12 hour light/dark cycle. Rats had ad lib access to food and water for the entire duration of this study, including abstinence period. Protocol approved by the Institutional Animal Care and Use Committee of the University of Toledo, Health Science Campus, Toledo, OH, was used for this study. The protocol was designed based on the guidelines set forth by the Institutional Animal Care and Use Committee of the National Institutes of Health and the Guide for the Care and Use of Laboratory Animals (Institute of Laboratory Animal Resources, Commission on Life Sciences, 1996). Before the start of the treatment (i.p.), animals were divided in three groups: 1) saline group (n=5); 2) ceftriaxone 50 mg/kg treatment group (n=5); 3) ceftriaxone 200 mg/kg treatment group (n=5). Ceftriaxone was administered as a solution made in physiological saline.

### Ethanol intake measurements

P rats at the age of 90 days old were given continuous access to two concentrations of ethanol solution, 15% and 30%, prepared with distilled water for 14 consecutive weeks. Multiple choices of ethanol solutions (15% and 30%) is an established model of ethanol drinking that is known to enhance ethanol intake in P rats [28,29]. Starting week 11, body weight, water intake, and ethanol consumption by P rats were recorded three times per week (Monday, Wednesday, and Friday). Ethanol and water measurements were taken to the nearest 10th of a gram by subtracting the weight of the bottle from its previous weight. The average of the data observed during weeks 13 and 14 for the three parameters- body weight, water intake, and ethanol consumption- served as the baseline value for the study. Importantly, animals with a baseline ethanol intake of less than 4 g/day were subsequently discarded from this study. After the completion of 14 weeks of ethanol consumption, animals were randomly divided into saline and ceftriaxone treatment groups and deprived of ethanol solutions for the next two weeks. During the last five days of the two-week abstinence period, saline or ceftriaxone was injected once daily around noon. We have chosen to treat the animals for 5 days based on previous studies from our lab and others demonstrating the effective of five-day treatment paradigm in upregulating the GLT1 levels in the key brain regions of the mesocorticolimbic reward pathway [23,25,30,31]. Subsequently, at the end of the two-week abstinence, this time point was also 24 h after the last dose of either saline or ceftriaxone, all P rats were re-exposed to the ethanol solutions and ethanol consumption, water intake, and body weights measurements were recorded daily for the next 10 days. On the 11th day, all animals were euthanized by exposure to CO_2_ inhalation.

### Statistical analyses

We used general linear model (GLM) repeated measures for statistical analysis (using SPSS statistical program) of data related to ethanol consumption, water intake, and P rat body weight. Furthermore, to observe the day-wise effect of treatment, data were analyzed employing one-way ANOVA for comparing the three treated groups using post hoc dunnett’s (two-sided) test with equal variances assumption since there was homogeneity of variance for ethanol and water intakes as well as body weight. All statistical tests were based on p<0.05 level of significance.

## Results

### Effect of ceftriaxone on ethanol intake

**Figure 1:**
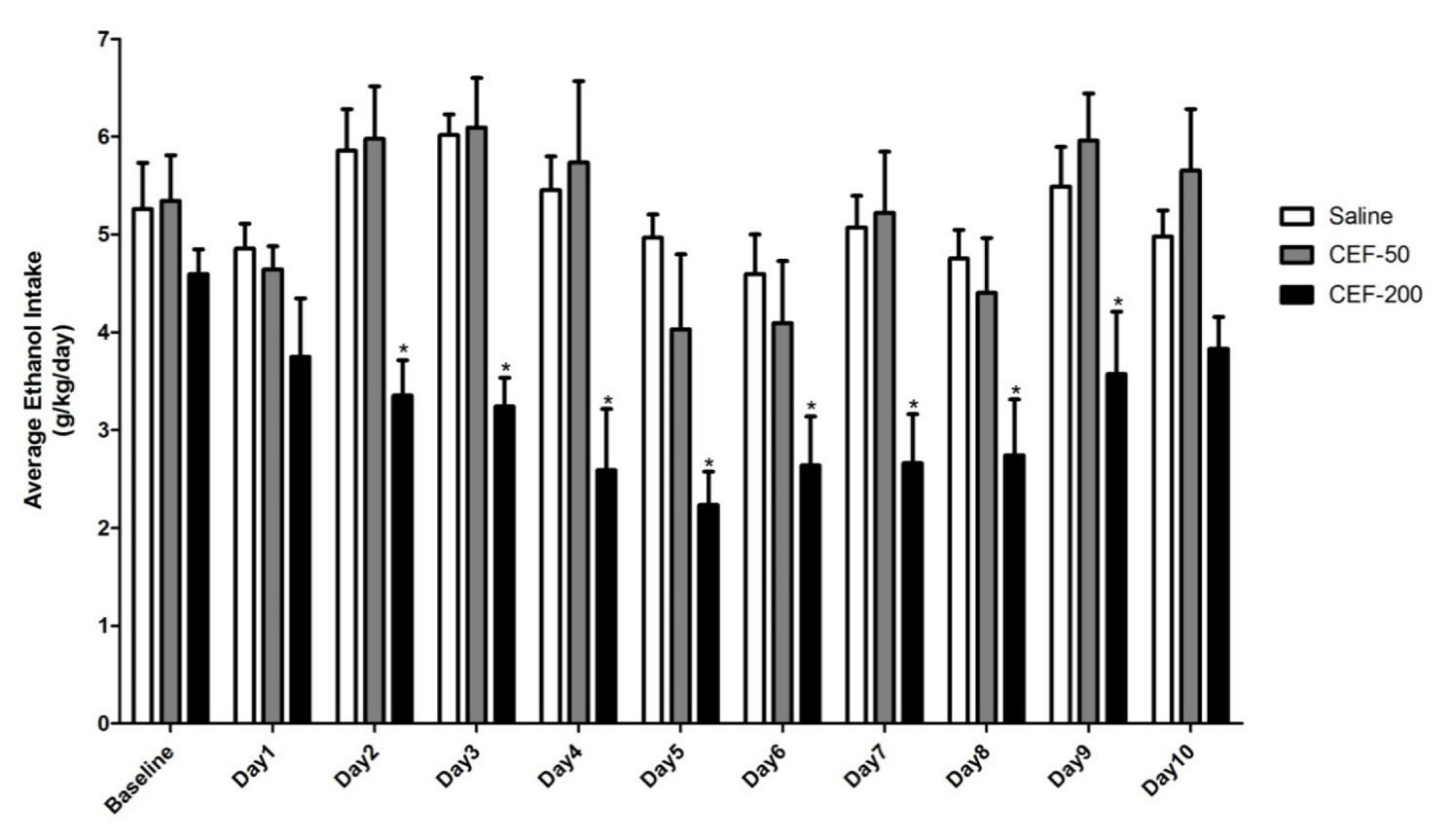
Graph represents average ethanol intake (g/kg/day) during the 10 days of re-exposure to ethanol. Based on GLM repeated measures followed by one-way ANOVA, ceftriaxone treatment (200 mg/kg/day) resulted in a significant reduction in ethanol consumption compared to saline vehicle-treated control group from Day 2 to Day 9. Lower dose of ceftriaxone (50 mg/kg/ day) did not cause any reduction in ethanol intake by P rats. Data are expressed as mean ± SEM (*: p<0.05). Saline group (n=5); ceftriaxone groups (50 and 100 mg/kg, i.p. body weight, n=6 for each group)

The effect of ceftriaxone treatment on relapse-like ethanol drinking behavior was monitored for 10 days following abstinence. Figure 1 represents the average ethanol consumption by P rats (g/kg/day) treated with either ceftriaxone (50 or 200 mg/kg/day) or saline vehicle. Baseline value represents the average ethanol consumed over the two weeks preceding abstinence (weeks 13 and 14). A GLM repeated measures analysis comparing ethanol consumption after re-exposure between the three groups revealed a significant main effect of Day [F(1,10)=7.65, p<0.05)] along with a significant Day X Treatment interaction effect [F(2,20)=2.11, p<0.05)]. One-way ANOVA, post hoc dunnett’s test, revealed that ceftriaxone treatment (during ethanol deprivation) induced reduction of ethanol intake upon re-exposure to ethanol, compared to saline treated group, was statistically significant (p<0.05) only for the higher dose (200 mg/kg). This effect was observed from Day 2 to Day 9. The lower dose of ceftriaxone used in this study (50 mg/kg/day) did not induce any significant decrease in ethanol consumption. These findings suggest that higher dose, which is known to upregulate GLT1 levels in key reward brain regions, has been effective in reducing ethanol intake.

### Effect of ceftriaxone on water consumption

**Figure 2:**
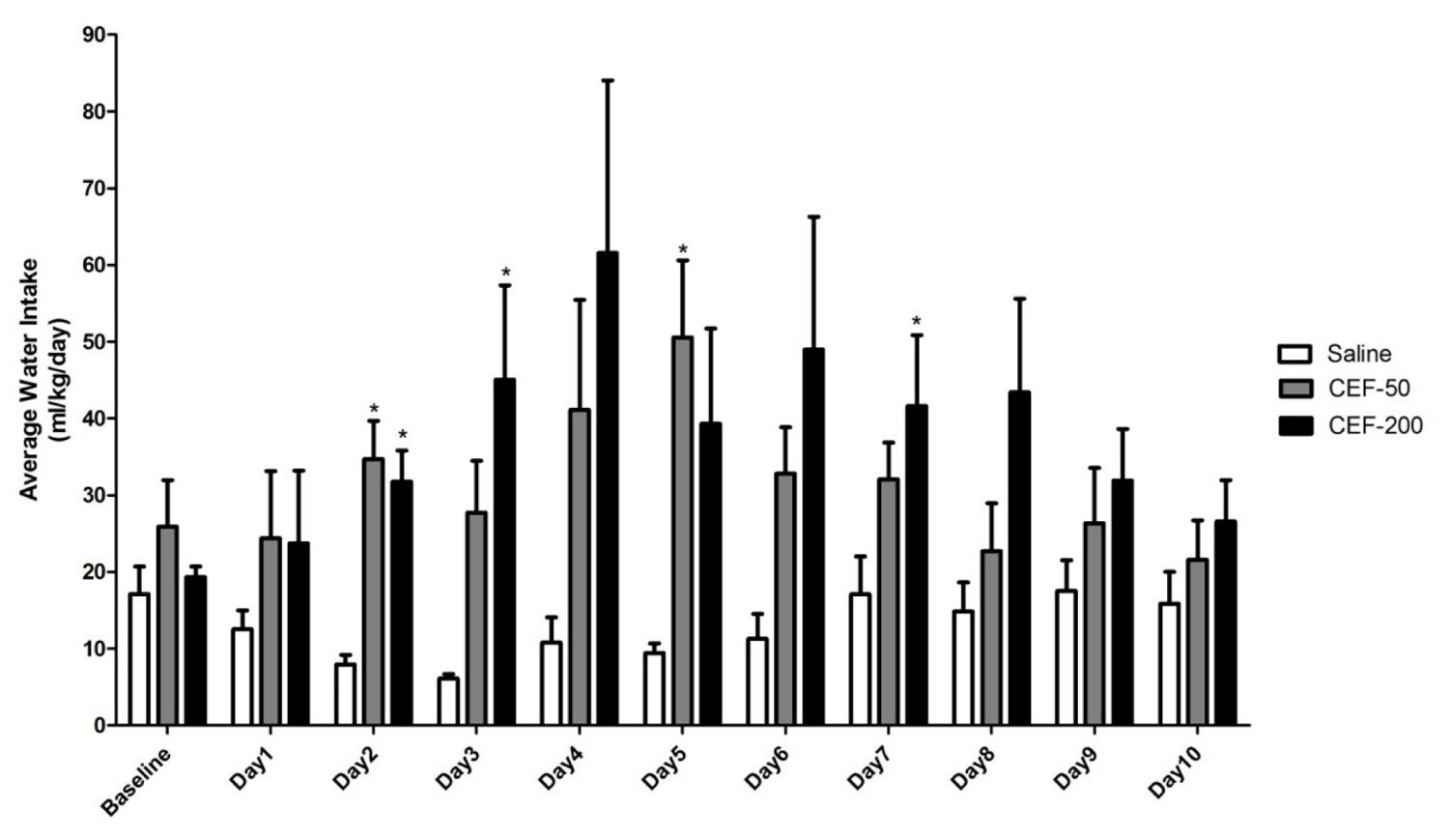
Graph represents average water intake (ml/kg/day) during the 10 days of re-exposure to ethanol. Based on GLM repeated measures followed by one-way ANOVA, ceftriaxone treatment resulted in significantly higher water consumption compared to saline-treated control group on Days 2 and 5 for 50 mg/kg, and on Days, 2, 3, and 7 for 200 mg/kg. Data are expressed as mean ± SEM (*: p<0.05). Saline group (n=5); ceftriaxone groups (50 and 100 mg/kg, i.p. body weight, n=6 for each group)

Following treatment with ceftriaxone, P rats consumed significantly higher amounts of water when compared to saline treated animals (Figure 2). A significant main effect of Day was observed [F(1,10)=3.53, p<0.05)] along with a significant Day X Treatment interaction effect [F(2,20)=2.93, p<0.05)]. Interestingly, one-way ANOVA revealed that water consumption was statistically different (p<0.05) in ceftriaxone (50 mg/kg/day) treated group as compared to saline treated group only on Day 2 and Day 5. Alternatively, animals treated with higher dose of ceftriaxone (200 mg/kg/day) consumed significantly higher amounts of water as compared to saline treated group, on Days 2, 3, and 7. These findings suggest that increase in water intake is considered as a compensatory mechanism for the decrease in ethanol intake, which is reflecting the amount body fluid intake.

### Effect of ceftriaxone on body weights

**Figure 3:**
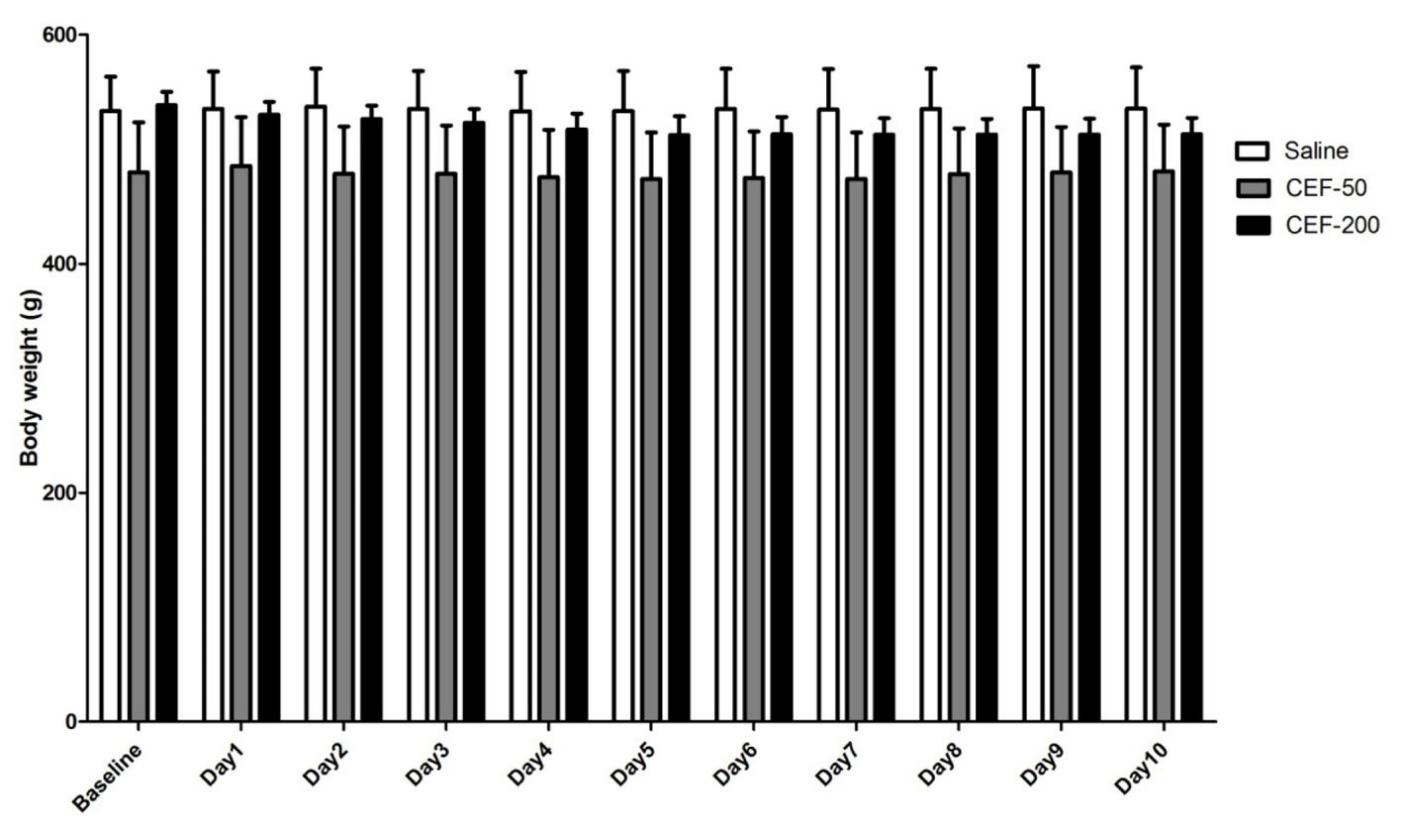
Graph represents average body weight (g) of P rats during the 10 days of re-exposure to ethanol. Based on GLM repeated measures followed by one-way ANOVA, ceftriaxone treatment (50 and 200 mg/kg/day) did not cause a significant change in body weights as compared to saline vehicle-treated control group. Data are expressed as mean ± SEM). Saline group (n=5); ceftriaxone groups (50 and 100 mg/kg, i.p. body weight, n=6 for each group)

A GLM repeated measures analysis revealed a significant main effect of Day [F(1,10)=6.83, p<0.05)] along with a significant Day X Treatment interaction effect [F(2,20)=3.98, p<0.05)] in body weights. One-way ANOVA did not reveal any significant differences in body weights between both ceftriaxone and saline treated groups (Figure 3). These findings suggest that higher dose did not induce any changes in food intake that may reflect the body weight of animals.

## Discussion

We report in this study that after long term continuous ethanol consumption (14 weeks) followed by two week deprivation, a dose dependent (50 vs. 200 mg/kg/day) effect was observed upon relapse-like ethanol drinking paradigm. During the 10 days of re-exposure to free-choice ethanol, the 200 mg/kg/day dose of ceftriaxone reduced ethanol intake from Day 2 to Day 9 compared to saline treated group. However, towards the end of the present study (Day 10), animals treated with the 200 mg/kg/day dose of ceftriaxone were found to consume amounts of ethanol equivalent to the saline-treated group. The lower dose (50 mg/kg/day) of ceftriaxone, did not change the ethanol drinking pattern following re-exposure.

These findings are in accordance with our previous studies that demonstrated that ceftriaxone at higher dose (200 mg/kg/day) attenuated reinstatement to cocaine-seeking behavior; however, the lower dose of ceftriaxone (50 mg/kg/day) did not induce any effect [30]. It is important to note that higher dose of ceftriaxone has been associated with upregulation of GLT1 in PFC and NAc [30].

Modulation of glutamatergic neurotransmission, including treatment with ceftriaxone, has been proved effective in attenuating relapse to drug addiction [24,25,27,32,33]. Studies investigated the underlying causes of alteration of glutamate neurotransmission have revealed that changes in the levels of two important proteins expressed on astrocytes, xCT [24,34] and GLT1 [23,25,31,35], are key players in drug abuse, including ethanol and cocaine.

Ceftriaxone and other compounds have been shown to modulate the glutamatergic neurotransmission primarily through the upregulation of GLT1 and xCT levels in the NAc in model of relapse to cocaine-seeking behavior [24,36]. Interestingly, administration of mGluR2/3 agonist and mGluR5 antagonist were found to attenuate cue-induced reinstatement of ethanol-seeking, which demonstrated the significance of glutamatergic neurotransmission in relapse to ethanol [37–39]. Our previous studies revealed the effectiveness of modulation of extracellular glutamate levels in the mesocorticolimbic pathway in relapse-like ethanol-drinking behavior [27]. After five weeks of ethanol exposure to P rats, ceftriaxone treatment was found to upregulate GLT1 in the NAc and PFC.

In the present study, although effective initially in reducing ethanol intake following re-exposure to ethanol in a 14 week relapse-like ethanol drinking paradigm, ceftriaxone (200 mg/kg/day) treatment did not maintain a statistically significant reduction in ethanol intake towards the end of the study period (Day 10). An explanation for the observed deviation of results compared to our previous study based on five-weeks of ethanol exposure can be found in the fact that changes in CNS neurotransmission are based on length of exposure [29]. Long-term exposure to ethanol is known to precipitate extensive modulation of the neurotransmitter systems [40–42]. The results from the present study are consistent with our recent finding that demonstrated the effects of ceftriaxone treatment after 14-weeks of continuous ethanol exposure [43]. After 14 weeks of continuous ethanol consumption, ceftriaxone induced reduction in ethanol intake, however, this effect diminished towards the end of the post-treatment period. In order to better understand the differences in ceftriaxone’s effectiveness based on length of ethanol exposure, further studies are warranted to examine the changes in neurotransmitter systems in 5-week versus 14- week ethanol-drinking paradigms.

Ceftriaxone treatment has been found effective in upregulating GLT1 levels in brain reward regions at 200 mg/kg, i.p. in rats [44,45]. This dose has been widely used in studies exploring the potential benefits, via GLT1 upregulation and subsequent modulation of glutamatergic neurotransmission, of ceftriaxone in attenuating drug dependence [46,47]. Importantly, while ceftriaxone treatment at higher doses attenuates the motivation for drug of abuse, it does not affect the responses for natural rewards, including sweet food [48]. Similarly, in our recent studies, we have shown that ceftriaxone treatment (200 mg/kg) does not affect the daily sucrose intake or body weights of P rats [23]. Therefore, based on the existing studies, this higher dose of ceftriaxone (200 mg/kg) was examined for its potential benefits in 14 week relapse-like ethanol-drinking model.

We conclude here that higher dose of ceftriaxone was effective in attenuating relapse-like ethanol-drinking behavior. However, the lower dose of ceftriaxone was ineffective. These findings are in accordance with our previous studies that revealed that higher dose of ceftriaxone, a dose known to upregulate GLT1 in PFC and NAc, was effective in attenuating reinstatement to cocaine-seeking behavior. Together, these findings suggest that the dose of ceftriaxone inducing upregulation of GLT1 is effective in the attenuation of relapse-like ethanol-drinking and cocaine-seeking behaviors.
